# Pulmonary Functional Outcomes at 3 Months in Critical COVID-19 Survivors Hospitalized during the First, Second, and Third Pandemic Waves

**DOI:** 10.3390/jcm12113712

**Published:** 2023-05-27

**Authors:** Cecile Dusart, Jelle Smet, Audrey Chirumberro, Stephanie André, Alain Roman, Marc Claus, Anne-Violette Bruyneel, Ophelie Menez, Stephane Alard, Nathalie De Vos, Marie Bruyneel

**Affiliations:** 1 Department of Pneumology, CHU Saint-Pierre, 1000 Brussels, Belgium; dusart.cec@gmail.com (C.D.); jelle.smet@azr.be (J.S.); audrey.chirumberro@stpierre-bru.be (A.C.); 2 Department of Pneumology, Université Libre de Bruxelles, CHU Saint-Pierre, 1000 Brussels, Belgium; stephanie.andre@chu-brugmann.be (S.A.); alain.roman@stpierre-bru.be (A.R.); marc.claus@stpierre-bru.be (M.C.); ophelie.menez@ulb.be (O.M.); stephane.alard@stpierre-bru.be (S.A.); nathalie.devos@lhub-ulb.be (N.D.V.); 3 Department of Pneumology, CHU Brugmann, 1020 Brussels, Belgium; 4 GDepartment of Intensive Care Medicine, CHU Saint-Pierre, 1000 Brussels, Belgium; 5 Geneva School of Health Sciences, HES-SO University of Applied Sciences and Arts Western Switzerland, 1206 Geneva, Switzerland; anne-violette.bruyneel@hesge.ch; 6 Department of Radiology, CHU Saint-Pierre, 1000 Brussels, Belgium; 7 Department of Clinical Chemistry, LHUB-ULB, CHU Saint-Pierre, 1000 Brussels, Belgium

**Keywords:** COVID-19, ARDS, chest CT, pulmonary function test, intensive care unit, quality of life, pandemic, wave, mortality

## Abstract

Introduction: Despite improved management of patients with COVID-19, we still ignore whether pharmacologic treatments and improved respiratory support have modified outcomes for intensive care unit (ICU) surviving patients of the three first consecutive waves (w) of the pandemic. The aim of this study was to evaluate whether developments in the management of ICU COVID-19 patients have positively impacted respiratory functional outcomes, quality of life (QoL), and chest CT scan patterns in ICU COVID-19 surviving patients at 3 months, according to pandemic waves. Methods: We prospectively included all patients admitted to the ICU of two university hospitals with acute respiratory distress syndrome (ARDS) related to COVID-19. Data related to hospitalization (disease severity, complications), demographics, and medical history were collected. Patients were assessed 3 months post-ICU discharge using a 6 min walking distance test (6MWT), a pulmonary function test (PFT), a respiratory muscle strength (RMS) test, a chest CT scan, and a Short Form 36 (SF-36) questionnaire. Results: We included 84 ARDS COVID-19 surviving patients. Disease severity, complications, demographics, and comorbidities were similar between groups, but there were more women in wave 3 (w3). Length of stay at the hospital was shorter during w3 vs. during wave 1 (w1) (23.4 ± 14.2 days vs. 34.7 ± 20.8 days, *p* = 0.0304). Fewer patients required mechanical ventilation (MV) during the second wave (w2) vs. during w1 (33.3% vs. 63.9%, *p* = 0.0038). Assessment at 3 months after ICU discharge revealed that PFTs and 6MWTs scores were worse for w3 > w2 > w1. QoL (SF-36) deteriorated (vitality and mental health) more for patients in w1 vs. in w3 (64.7 ± 16.3 vs. 49.2 ± 23.2, *p* = 0.0169). Mechanical ventilation was associated with reduced forced expiratory volume (FEV1), total lung capacity (TLC), diffusing capacity for carbon monoxide (DLCO), and respiratory muscle strength (RMS) (w1,2,3, *p* < 0.0500) on linear/logistic regression analysis. The use of glucocorticoids, as well as tocilizumab, was associated with improvements in the number of affected segments in chest CT, FEV1, TLC, and DLCO (*p* < 0.01). Conclusions: With better understanding and management of COVID-19, there was an improvement in PFT, 6MWT, and RMS in ICU survivors 3 months after ICU discharge, regardless of the pandemic wave during which they were hospitalized. However, immunomodulation and improved best practices for the management of COVID-19 do not appear to be sufficient to prevent significant morbidity in critically ill patients.

## 1. Introduction

Coronavirus disease 2019 (COVID-19) is the third and most important outbreak of coronavirus this century. This outbreak was recognized as a pandemic by the World Health Organization on the 11th of March, 2020. As of the 28th of January, 2022, among a total population of approximately 364 million who have contracted SARS coronavirus-2 (SARS-CoV-2), more than 5.63 million people have died worldwide [1]. The number of patients affected by SARS-CoV-2 is still rising, with striking problems related to emerging variants of concern (VOC) despite vaccination rates of up to 84% in European countries [2].

The spectrum of disease is broad, with 5% of symptomatic COVID-19 patients suffering from severe respiratory failure, fulfilling the Berlin definition of acute respiratory distress syndrome (ARDS), requiring intensive care unit (ICU) admission [3,4]. Despite the development of a panel of treatments that have shown efficacy for improving patient outcomes and reducing mortality (e.g., anti-coagulation, glucocorticoids (GC), anti-viral therapies, monoclonal antibodies, ventilatory support) [5,6], we still face, in ICU-admitted patients, multi-organ acute complications related to SARS-CoV-2 infection (renal, hepatic, thromboembolic, neurologic, cardiac, muscular). These may be directly attributable to the virus, such as immune-mediated mechanisms or microangiopathy, or indirect challenges related to subsequent long hospital stays, bed rest, iatrogenic factors, or psychological disorders [7,8,9]. We do not yet know whether the present management of ICU COVID-19 patients has changed long-term outcomes compared to patients who were initially affected at the start of the pandemic.

The aim of the present study was to evaluate whether developments in the management of ICU COVID-19 patients have positively impacted outcomes in ICU COVID-19 survivors 3 months after ICU discharge, according to the timing of hospitalization (1st, 2nd, and 3rd pandemic waves). We hypothesized that pulmonary function recovery would be better in patients treated in wave 3 compared to waves 1 and 2. Outcomes included comparisons of pulmonary function test (PFT), 6 min walking distance test (6MWT), respiratory muscle strength (RMS) test, quality of life (QoL), and radiologic assessment 3 months after ICU discharge. Predictors of poor outcomes were also analyzed.

## 2. Methods

### 2.1. Design

This was a prospective observational and multicentric study. Patients admitted to intensive care for COVID-19-related ARDS at CHU Saint-Pierre and CHU Brugmann (tertiary reference hospitals, Brussels, Belgium) were included in the study 3 months from ICU discharge during the first three pandemic waves.

### 2.2. Patients

Adult patients were included at 3 months from ICU discharge and assessed using chest computed tomography (CT), lung function tests, and questionnaires. All ICU COVID-19 patients were eligible as long as the reason for admission to the ICU was acute respiratory failure due to COVID-19 and not another pathology with an incidental finding of associated COVID-19. Exclusion criteria included language barrier, refusal, and psychiatric or mental disorders.

All included patients provided written informed consent to participate in the study. The study protocol was approved by the Saint-Pierre University Hospital ethics committee (AK/16-01-18/4613) and by the Brugmann University Hospital ethics committee (CE2020/141). All procedures performed in studies involving human participants were in accordance with the ethical standards of the institutional and/or national research committee and with the 1964 Helsinki Declaration and its later amendments or comparable ethical standards.

### 2.3. Data Collection

#### 2.3.1. Baseline Data

Data related to hospitalization and medical history were collected from the medical files of the patients including demographics, anthropometric data, comorbidities, toxic habits, SARS-CoV-2 vaccination status, APACHE II score, Sequential Organ Failure Assessment (SOFA) score at ICU admission, biological data, COVID-19 treatments, and complications.

The first COVID-19 pandemic wave (w1) refers to ICU admission between 1 March 2020 and 9 October 2020 (predominant VOC: alpha), the second wave (w2) was between 10 October 2020 and 14 March 2021 (predominant VOC: delta), and the third wave (w3) was between 15 March 2021 and 30 June 2021 (predominant VOC: delta).

#### 2.3.2. 3-Month Assessment

A comprehensive assessment of patients was performed 3 months after ICU discharge, according to a procedure previously described by Truffaut et al. [10]. 

PFTs, including spirometry, body plethysmography, and diffusing capacity for carbon monoxide (DLCO), were performed on the Carefusion MasterScreen body diff RT with SentrySuite software. The following values were analyzed: total lung capacity (TLC; in liters and percent predicted value), forced vital capacity (FVC) (in liters and percent predicted value), forced expiratory volume of 1s (FEV1) (in liters and percent predicted value), Tiffeneau Index (FEV1/FVC), DLCO using single breath test in mmol/minute/kilo Pascal (in percent predicted value, corrected for hemoglobin). GLI reference values were used [11,12]. A 6 min walking distance test (6MWT) with continuous peripheral oxygen saturation monitoring was performed [13]. 

Respiratory muscle strength (RMS) measurements to evaluate Maximum Inspiratory and Expiratory Mouth Pressures (MIP/MEP) were done with the MicroRPM (Micro Medical) according to international recommendations [14].

Assessments also included three questionnaires. QoL was assessed using the Short Form 36 (SF-36) [15]. The 36 questions assessed 8 dimensions of function and well-being, with lower scores corresponding to more disability. Breathlessness was evaluated using the modified Medical Research Council dyspnea scale (mMRC), where more points indicate more dyspnea. Finally, post-COVID disability was assessed using the post-COVID Functional Status (PCFS) test [16]. This is an ordinal scale established by Klok et al. at the beginning of the pandemic for determining functional recovery from COVID-19, where more points indicate more disability. 

Chest CT scans were reviewed by a single senior radiologist with extensive experience (>20 years). Reported Chest CT abnormalities were ground glass opacities, consolidation and fibrosis (including bronchiolectasis, fibrotic strands, irregular lines, and reticulations).

### 2.4. Statistical Analysis

The statistical data treatment included descriptive statistics. Qualitative data are expressed as frequencies and percentages. For quantitative data, the mean and standard deviation were calculated. 

The demographic, clinical characteristic, and clinical outcomes data were compared between the three waves (w1 vs. w2 vs. w3). Normality and homoscedasticity were assessed with Shapiro’s test and Levene’s test, respectively, for quantitative variables. In the case of data with a normal distribution, a one-way ANOVA was calculated. In cases where the data were not normally distributed, the non-parametric Kruskal–Wallis test was used with a Dunn post-hoc test. The comparison between the three waves for qualitative data was tested with the Marascuilo procedure (binary outcomes—yes/no) or Kruskal–Wallis (ordinal data). The Marascuilo procedure was used to compare proportions when there were more than two independent groups. This was initially used to perform a test of overall homogeneity for a large contingency table, using the standard Chi-square Test. This was followed by multiple post-hoc comparisons between pairs of groups in the data. 

For each wave, the predictive factors of the 3-month clinical outcomes were assessed. A multiple linear or logistic regression analysis (according to data type) was used to determine the variables that had the greatest influence.

A *p*-value below 0.05 was considered statistically significant. All analyses were performed using Python (version 3.8) with the statistics package statsmodels (version 0.11.1). The package was released under the open source modified BSD (3-clause) license.

### 2.5. Results

Eighty-four ARDS survivors of the first three COVID-19 pandemic waves were included in the study. The relatively low number of included patients was related to language barriers, refusal, or follow-up in other hospitals. Indeed, during the first three waves of the pandemic, many patients were transferred from other regions of Belgium or even from abroad due to a lack of beds. As a result, a number of patients returned to their region of origin for post-hospitalization follow-up. 

Disease severity, complications, demographics, and comorbidities were similar between groups, but there were more women in w3. No patients were vaccinated against SARS-CoV-2. The ICU mortality rate was not significantly different between w1 and w2 (29% and 36%, *p* = 0.2900) but was significantly lower during w3 vs. during w1 (18% vs. 29%, *p* = 0.0500) and w2 (18% vs. 36%, *p* = 0.0002). Demographics and clinical data for the 84 included patients are summarized in Table 1. 

Mean hospital length of stay was shorter during w3 vs. during w1 (23.4 ± 14.2 days vs. 34.7 ± 20.8 days, *p* = 0.0304). Fewer patients required mechanical ventilation (MV) during w2 vs. during w1 (33.3% vs. 63.9%, *p* = 0.0038), such that the use of curare was also reduced (*p* = 0.0227). The use of tocilizumab increased significantly during w3 (71.4% vs. 19.4% for w1, *p* < 0.0001 and vs. 3.7% for w2, *p* < 0.0001) while broad-spectrum antibiotic use decreased.

The 3-month evaluation took place after a median of 91 days (min–max 73–125).

Three months after ICU discharge, results of PFTs, RMS tests, and 6MWTs did not improve in w2 and w3 compared to w1. Patients from w2 demonstrated a reduced TLC (% predicted value) compared to w1 (*p* = 0.0094). QoL, assessed using the SF-36, was worse in the vitality and mental health items for patients from w1 vs. patients from w3 (total scores 64.7 ± 16.3 vs. 49.2 ± 23.2, *p* = 0.0169). These results are summarized in Table 2.

No differences in affected segments were observed at baseline and 3-month chest CT, but at three months, more patients from w2 vs. w1 exhibited ground glass opacities (41% vs. 3%, *p* = 0.0006) and fibrosis was much more frequent in w1 vs. w2 (94% vs. 67%, *p* = 0.0186, Table 3). 

The multiple linear or logistic regression analysis highlighted that MV was associated with reduced TLC, FEV1, DLCO (all waves), reduced maximum inspiratory pressure (PI Max) (w1 and w2), and 6MWT (w1 and w3) at 3 months (*p* < 0.0229) (Table 4).

The use of GC was associated with better TLC, FEV1, DLCO, and a lower number of affected segments on 3-month CT (all *p* < 0.0001) (Table 4). 

Moreover, tocilizumab was associated with better TLC, FEV1, DLCO, and a lower number of affected segments on 3-month CT (all *p* < 0.0058, Table 4). 

## 3. Discussion

This study shows that, despite progressively better understanding and management of COVID-19 patients, pulmonary functional outcomes (PFT, 6MWT, RMS) did not improve in the three first waves of the pandemic. No positive change in outcomes was observed during the second and third waves compared to the first. 

Upon multivariate analysis, the main factor explaining pulmonary functional outcomes was MV, which reflects more severe illness. Obviously, the more severe patients exhibit worse short- and medium-term lung status [17]. Several studies have focused on ICU mortality rates and management differences between waves. Carbonell et al. reported, as in the present series, an increased use of GC in w2/3 vs. in w1 [18]; the need for MV remained high in the three waves but an increased use of high-flow nasal oxygen (HFNO) was described that was not observed in our survivors. In their study, mortality was similar during the first three consecutive waves, whereas it decreased significantly in our series. In a Swiss study comparing short-term outcomes of hospitalized patients during the first and second waves, mortality was also similar despite the use of GC in 76% vs. 0% of patients [19]. Length of stay was shorter by 2.5 days in w2, as observed in our series for w3 vs. w1 (-11 days). 

Our study particularly focused only on early outcomes in ICU COVID-19 survivors. Outcomes in hospitalized, including ICU, patient cohorts from w1 at 3–6 months are now well documented [10,17,20,21,22,23], showing alterations in PFT (restrictive +/− decreased DLCO) and 6MWT in about half of the patients, radiologic abnormalities in more than 70% of patients, and dyspnea in one-third. Radiologic abnormalities have been correlated with the length of MV [19], length of ICU stay [20], initial radiologic extension [10], ICU admission, and MV [21]. However, no studies have compared the outcomes of hospitalized patients according to successive pandemic waves.

It is surprising and disappointing that increased experience and use of effective medications in critically ill patients since the mid-first wave did not change outcomes in survivors, despite a proven impact on 28-day mortality for GC [5], tocilizumab, and remdesivir when given at the right time to the right patient [24,25]. Outcomes are probably more related to ARDS and ICU stay complications than to specific medications or ventilatory support. However, the present study lacks a larger reference population (those who were diagnosed with COVID-19 in the general population or admitted to the hospital with COVID-19 and not admitted to an ICU). In this context, we can suppose that improved therapies for COVID-19 management prevented patients from being admitted to the ICU, but in cases where unfavorable clinical evolution required intensive care, the clinical evolution in survivors was identical, whatever the wave of the pandemic.

Mechanical ventilation is still a factor related to poor outcomes 3 months after discharge, regardless of the pandemic wave. MV has been reported as a major risk factor for death by COVID-19 in two European studies [18,26], but when they survive, patients are also more disabled after MV. Impaired DLCO and 6MWT have been shown to be related to MV in a Spanish series of 78 COVID-19 ICU patients [26]. Noel-Savina et al., in a French cohort of 72 patients that included 75% ICU COVID-19 patients, also showed that COVID-19 patients who required MV exhibited more frequently impaired DLCO, gas exchange abnormalities, and interstitial lung disease on chest CT scan 4 months after discharge [22]. Lorent et al., in a mixed Belgian cohort of 299 hospitalized patients with moderate and severe COVID-19, also showed that impaired DLCO, TLC, 6MWT, and quadriceps strength was significantly more reduced in severe than in moderate patients. Chest CT severity score was also higher in these severe patients, of which 77% were intubated [23].

We have also noted, in our cohort, that results of PFT (some components) and 6MWT (% predicted value (PV)) were worse for w3 > w2 > w1. The w3 population consisted of more women who were more often obese. Obesity can potentially affect both results. Indeed, FEV1 and FVC (in liters) were lower in w3 patients, but when results were expressed in PV, they were not different, suggesting that this observation was only related to gender/height. Of note, lung volume reduction in obese women is rarely observed. In a cohort reported by Buyse et al. [27] where 117 obese women were studied, mean BMI was 43 and all lung volumes were normal. On the 6MWT, distance expressed in PV was lower for w3 > w2 > w1 (but statistically non-significant). The ability to perform this test is reduced in obese individuals [28] and the fact that a majority of patients from w3 were obese may have strongly influenced the results of this test in this group.

However, despite our results, we can be optimistic about the ability of critically ill COVID-19 patients to recover; longitudinal cohort studies have shown that patients improve significantly between 3 months and 1 year [23,29]. 

## 4. Limitations

Despite the relatively small sample size of our study, our results appear to accurately reflect the outcomes for the critically ill COVID-19 population, as patient characteristics and management in this study were similar to other larger series [17,18,21]. Another limitation is the absence of pre-ICU management details. However, starting May 25 (w1), all had received GC and (high-flow) oxygen before ICU admission. Therefore, even though the patients were similar in terms of disease severity, age, BMI, and comorbidities, we have compared groups of different patients, heterogeneous by definition.

## 5. Conclusions 

The present study shows, for the first time, a comparison of pulmonary functional outcomes, radiologic impairment, and QoL 3 months after ICU discharge in a small sample of critically ill COVID-19 patients in the three waves of the COVID-19 pandemic. Disappointingly, despite increased experience and the use of more effective medications since the mid-first wave of COVID-19, PFT, 6MWT, RMS, and chest CT alterations remained similar overall (and certainly not better over time) in ICU survivors, regardless of the wave of the pandemic in which they were hospitalized. Immunomodulation and current best practices for the management of COVID-19 do not appear to be sufficient to prevent significant morbidity in critically ill patients. More research is needed to improve the ICU management of COVID-19 and, more importantly, to prevent admissions to the ICU through improved knowledge about the best early treatment of COVID-19.

## Figures and Tables

**Table 1:** Baseline characteristics of patients, according to the wave of the pandemic.

Variables (Mean, SD, or %)	Wave 1	Wave 2	Wave 3	*p*-Value
	n = 36	n = 27	n = 21	
Age (years)	56.64 ± 10.33	59.48 ± 10.92	53.1 ± 13.06	0.1555
Sex, Women	n = 8 (22.2%)	n = 11 (14.7%)	n = 12 (57.1%)	0.0274
BMI > 30	n = 22 (61.1%)	n = 14 (51.9%)	n = 20 (95.2%)	0.5739
Current smokers	n = 13 (36.11%)	n = 7 (25.92%)	n = 8 (38.09%)	0.6047
Medical history				
Cancer	n = 1 (2.78%)	n = 1 (3.70%)	n = 1 (2.78%)	0.9261
Diabetes	n = 15 (41.67%)	n = 15 (55.56%)	n = 8 (38.10%)	0.4111
Hypertension	n = 20 (55.56%)	n = 17 (62.96%)	n = 10 (47.62%)	0.5677
HIV	n = 2 (5.56%)	n = 0	n = 0	N/A
Obstructive sleep apnea	n = 6 (16.67%)	n = 1 (3.70%)	n = 2 (9.52%)	2526
Length of stay (days)				
Hospital	34.72 ± 20.76	27.81 ± 16.93	23.43 ± 14.25	0.6097
ICU	20.5 ± 15.97	15.04 ± 14.03	16.95 ± 11.72	0.7744
Discharge to rehabilitation unit	n = 8 (22.2%)	n = 8 (29.6%)	n = 5 (23.8%)	0.1895
ICU severity scores				
APACHE	10.06 ± 4.05	9.04 ± 4.82	7.57 ± 5.02	0.3045
SOFA	3.67 ± 2.29	3.11 ± 1.25	3.62 ± 1.69	0.1185
RESPIRATORY				
SUPPORT *				
Mech. ventilation	n = 23 (63.89%)	n = 9 (33.33%)	n = 10 (47.62%)	0.0543
mean duration (days)	12.22 ± 13.44	4.67 ± 7.58	7.86 ± 9.82	0.1099
curare	n = 21 (58.33%)	n = 7 (25.93%)	n = 9 (42.86%)	0.0371
prone	n = 16 (44.44%)	n = 10 (37.04%)	n = 8 (38.09%)	0.8117
ECMO	n = 9 (25.00%)	n = 8 (29.63%)	n = 1 (4.76%)	0.09
High flow oxygen	n = 3 (8.33%)	n = 1 (3.70%)	n = 3 (14.29%)	0.4207
CPAP	n = 0	n = 0	n = 4 19.05(%)	N/A
BIPAP	n = 2 (5.56%)	n = 9 (33.33%)	n = 6(28.57%)	0.0137
Laboratory data				
D-dimer, mg/dL	9137.57 ± 10,440.87	4436.48 ±6939.15	6993.19 ± 9353.9	0.1408
CRP, mg/dL	244.52 ± 106.17	174.03 ± 95.62	165.0 ± 94.07	0.5058
Treatments				
glucocorticoids	n = 21 (58.33%)	n = 27 (100%)	n = 21 (100%)	N/A
favipiravir	n = 3 (8.33%)	n = 0	n = 0	N/A
remdesivir	n = 3 (8.33%)	n = 2 (7.41%)	n = 0	N/A
monoclonal antibody	n = 1 (2.78%)	n = 0	n = 0	N/A
tocilizumab	n = 7 (19.44%)	n = 1 (3.70%)	n = 15 (71.43%)	<0.0001
broad-spectrum antibiotics	n = 31 (86.11%)	n = 23 (85.19%)	n = 11 (52.38%)	0.0067
Complications				
Thromboembolic event	n = 12 (33.33%)	n = 5 (18.52%)	n = 3 (14.29%)	0.1953
Critical illness polyneuropathy	n = 3 (8.3%)	n = 3 (11.1%)	n = 4 (19%)	0.4781
Atrial fibrillation	n = 1 (2.7%)	n = 1 (3.7%)	n = 0	N/A

*: some patients received more than one modality (consecutive therapies). ICU: intensive care unit, ECMO: extracorporeal membranous oxygenation, CPAP: continuous positive airway pressure, BIPAP: bilevel positive airway pressure, CRP: C-reactive protein.

**Table 2:** Comparisons of clinical outcomes between waves.

Variables (Mean, SD or %)	Wave 1	Wave 2	Wave 3	*p*-Value
	n = 36	n = 27	n = 21	
PFT				
FEV1 (L)	2.88 ± 0.72	2.47 ± 0.82	2.26 ± 0.74	0.0096
FEV1 (%)	87.92 ± 14.71	79.81 ± 21.06	79.19 ± 17.67	0.1056
FVC (L)	3.41 ± 0.83	2.95 ± 0.96	2.8 ± 0.94	0.0322
FVC (%)	82.28 ± 15.73	74.52 ± 19.58	78.33 ± 18.25	0.2299
FEV1/FVC	0.83 ± 0.07	0.83 ± 0.08	0.82 ± 0.1	0.2218
TLC (L)	5.61± 1.28	4.81± 1.37	4.49± 1.2	0.0048
TLC (%)	87.97 ± 15.02	75.59 ± 21.3	83.43 ± 21.2	0.042
DLCO (%)	81.12 ± 18.32	78.24 ± 22.23	75.35 ± 22.34	0.6118
PFT interpretation				0.5868
normal	n = 14 (38.8%)	n = 7 (25.9%)	n = 10 (47.6%)	
restrictive pattern	n = 7 (19.4%)	n = 7 (25.9%)	n = 3 (14.2%)	
restrictive + decreased DLCO	n = 6 (16.7%)	n = 6 (22.2%)	n = 4 (19%)	
isolated decreased DLCO	n = 9 (25%)	n = 7 (25.9%)	n = 4 (19%)	
6MWT	n = 34	n = 26	n = 20	
distance (m)	500.18 ± 89.07	417.71 ± 132.89	434.1 ± 109.0	0.025
distance (%)	73.05 ± 11.36	83.67 ± 91.36	67.88 ± 16.45	0.1831
oxygen desaturation	n = 11 (33.33%)	n = 6 (24.00%)	n = 6 (30.00%)	0.7411
RMS	n = 34	n = 26	n = 21	
IP max (cm H20)	89.03 ± 28.06	70.98 ± 34.01	77.81 ± 35.9	0.0931
EP max (cm H20)	105.95 ± 32.06	87.71 ± 39.09	85.57 ± 36.07	0.0637
mMRC	n = 34	n = 27	n = 21	0.0517
0	n = 18 (52.94%)	n = 6 (22.22%)	n = 7 (33.33%)	
1	n = 10 (29.41%)	n = 13 (48.15%)	n = 4 (19.05%)	
2	n = 3 (8.82%)	n = 5 (18.52%)	n = 7 (33.33%)	
3	n = 3 (8.82%)	n = 1 (3.70%)	n = 2 (9.52%)	
4	n = 0	n = 2 (7.41%)	n = 1 (4.76%)	
PCFS (%)	n = 33	n = 25	n = 21	0.7731
0	n = 4 (30.77%)	n = 5 (20.00%)	n = 5 (23.81%)	
1	n = 4 (30.77%)	n = 9 (36.00%)	n = 6 (28.57%)	
2	n = 2 (15.38%)	n = 4 (16.00%)	n = 5 (23.80%)	
3	n = 3 (23.08%)	n = 6 (24.00%)	n = 1 (4.76%)	
4	n = 0	n = 1 (4.00%)	n = 4 (19.05%)	
SF-36	n = 34	n = 26	n = 21	
Global score	64.71 ± 16.26	57.23 ± 21.46	49.19 ± 23.24	0.0241
Physical functioning	68.03 ± 24.01	61.15 ± 29.27	52.62 ± 28.44	0.129
Role-Physical	39.39 ± 39.05	27.88 ± 40.2	46.9 ± 36.59	0.2401
Pain	65.48 ± 31.04	58.87 ± 33.56	55.29 ± 33.56	0.5052
General health	60.76 ± 16.97	56.77 ± 20.11	51.05 ± 19.34	0.1826
Vitality	57.42 ± 17.33	45.96 ± 22.76	39.52 ± 24.18	0.0089
Social	75.38 ± 21.98	68.75 ± 30.67	60.71 ± 32.9	0.1786
Role-Emotionnal	46.47 ± 45.6	51.28 ± 45.44	38.09 ± 42.54	0.6026
Mental health	70.3 ± 16.43	64.31 ± 22.96	51.24 ± 21.19	0.0042

PFT: pulmonary function test, FEV1: forced expiratory volume in 1s, FVC: forced vital capacity, TLC: total lung capacity, DLCO: diffusing capacity for carbon monoxide, 6MWT: 6 min walking distance test, RMS: respiratory muscle strength, SF-36: Short Form 36, PCFS: post-COVID Functional Status, mMRC: modified Medical Research Council dyspnea scale, IP: inspiratory pressure, EP: expiratory pressure, oxygen desaturation: desaturation ≥ 4% during the 6MWT.

**Table 3:** Comparison of radiological characteristics between waves.

Chest CT Scan	Wave 1	Wave 2	Wave 3	*p*-Value
	N = 36	N = 27	N = 21	
number affected segments (mean +/− SD)				
● Baseline	16.29 ± 4.27	17.85 ± 3.81	16.67 ± 4.32	0.3275
● 3-month	9.19 ± 6.74	10.56 ± 8.04	8.14 ± 6.66	0.7378
3-month Chest CT observations				
ground glass opacities	n = 1 (2.78%)	n = 11 (40.74%)	n = 4 (19.05%)	0.0007
consolidation	n = 4 (11.11%)	n = 3 (11.11%)	n = 4 (19.05%)	0.6467
fibrosis	n = 34 (94.44%)	n = 18 (66.67%)	n = 15 (71.43%)	0.0137

**Table 4:** Multiple regression results for mechanical ventilation and glucocorticoid and tocilizumab treatments.

Predictive Variables	Dependent Variables	Wave with Significant Results	R	R2	R2 Adjusted	*p*-Value
Mechanical ventilation	TLC	w1, w2, w3	≥0.93	≥0.87	≥0.86	≤0.0229
	FEV1	w1, w2, w3	≥0.94	≥0.88	≥0.87	≤0.0060
	DLCO	w1, w2, w3	≥0.92	≥0.84	≥0.82	≤0.0200
	IP Max	w1, w2	≥0.91	≥0.82	≥0.78	≤0.0175
	6MWT	w1, w3	≥0.93	≥0.87	≥0.84	≤0.0107
Glucocorticoids	TLC	w1, w2, w3	≥0.70	≥0.49	≥0.47	≤0.0001
	FEV1	w1, w2, w3	≥0.70	≥0.49	≥0.47	≤0.0001
	DLCO	w1, w2, w3	≥0.70	≥0.49	≥0.47	≤0.0001
	3-month CT	w1, w2, w3	≥0.70	≥0.50	≥0.48	≤0.0001
Tocilizumab	TLC	w1, w3	≥0.50	≥0.25	≥0.22	≤0.0021
	FEV1	w1, w3	≥0.47	≥0.22	≥0.20	≤0.0035
	DLCO	w1, w3	≥0.50	≥0.25	≥0.22	≤0.0027
	3-month CT	w3	0.57	0.32	0.29	0.0058

FEV1: forced expiratory volume in 1s, TLC: total lung capacity, DLCO: diffusing capacity for carbon monoxide, IP: inspiratory pressure, 6MWT: 6 min walking distance test, 3-month CT: number of affected segments on 3-month chest CT scan.

## Data Availability

The datasets used and analyzed during the current study are available from the corresponding author upon reasonable request.
